# Application of CRISPR/Cas9 system to knock out *GluB* gene for developing low glutelin rice mutant

**DOI:** 10.1186/s40529-024-00432-0

**Published:** 2024-09-03

**Authors:** Latifa AlHusnain, Muneera D. F. AlKahtani, Kotb A. Attia, Tayyaba Sanaullah, Dalia E. Elsharnoby

**Affiliations:** 1 Department of Biology, College of Science, Princess Nourah bint Abdulrahman University, P.O. Box 84428, Riyadh, 11671 Saudi Arabia; 2 Center of Excellence in Biotechnology Research, King Saud University, P.O. Box2455, Riyadh, 11451 Saudi Arabia; 3 Department of Botany, Government Sadiq College Women University, Bahawalpur, 53100 Pakistan; 4 Rice Research and Training Center, Field Crops Research Institute, Agricultural Research Center, Kafrelsheikh, 33717 Egypt

**Keywords:** Low glutelin rice, Amino acid composition, Irregularly-shaped protein bodies II, Mutation breeding, Nutritional quality

## Abstract

The nutritional quality improvement is among the most integral objective for any rice molecular breeding programs. The seed storage proteins (SSPs) have greater role to determine the nutritional quality of any cereal grains. Rice contains relatively balanced amino acid composition and the SSPs are fractioned into albumins (ALB), globulins (GLO), prolamins (PRO) and glutelins (GLU) according to differences in solubility. GLUs are further divided into subfamilies: GluA, GluB, GluC, and GluD depending on resemblance in amino acid. The GLU protein accounts for 60–80% of total protein contents, encoded by 15 genes located on different chromosomes of rice genome. The clustered regularly interspaced short palindromic repeats (CRISPR)/CRISPR-associated protein 9 (Cas9) system was employed to knockout *Glu-B* (*LOC-Os02g15070*) gene in non-basmati rice PK386 cultivar. The mutant displayed two base pair and three base pair mutation in the targeted regions. The homozygous mutant plant displayed reduction for both in total protein contents and GLU contents whereas, elevation in GLO, ALB and PRO. Moreover, the mutant plant also displayed reduction in physio-chemical properties e.g., total starch, amylose and gel consistency. The agronomic characteristics of both mutant and wild type displayed non-significant differences along with increase in higher percentage of chalkiness in mutant plants. The results obtained from scanning electron microscopy showed the loosely packed starch granules compared to wild type. The gene expression analysis displayed the lower expression of gene at 5 days after flowering (DAF), 10 DAF, 15 DAF and 20 DAF compared to wild type. GUS sub-cellular localization showed the staining in seed which further validated the results obtained from gene expression. Based on these findings it can be concluded *Glu-B* gene have significant role in controlling GLU contents and can be utilized in breeding programs to enhance the nutritional quality of rice, and may serve as healthy diet for patient allergic with high GLU contents.

**Supplementary Information:**

The online version contains supplementary material available at 10.1186/s40529-024-00432-0.

## Introduction

Seed storage proteins (SSPs) are deposited nitrogen source essential for seed germination along with nutrient source for humans and livestock. Since, the seed contains the substantial amount of SSPs and determine the nutritional quality (Shewry and Halford 2002). The nutritional quality improvement is among the most integral objective any rice molecular breeding programs (Zafar and Jianlong, 2023); however, SSPs genetic architecture is controlled by multigene families, reducing phenotypic alternation in crude protein content and amino acid composition caused by several mutations in few structural genes. The rice seed storage proteins include acid/alkaline-soluble glutelins, alcohol-soluble prolamins, and saline-soluble α-globulin (Oparka and Harris 1982).

The rough endoplasmic reticulum (ER) is key place for the synthesis of SSPs, translocated to ER lumen and further transferred to discrete intracellular compartments of plant’s endomembrane system (Li et al. 1993). Rice contains relatively balanced amino acid composition and the SSPs are fractioned into albumins (ALB), globulins (GLO), prolamins (PRO) and glutelins (GLU) according to differences in solubility (Shewry 1995). In most cereals, PRO are the major proteins, whereas oat (*Avena sativa*) preferentially accumulate SSPs belonging to the 11 S and 12 S type GLO family, rice (*Oryza sativa*) accumulate GLU (Shewry and Halford 2002). In rice, the GLU protein accounts for 60–80% of total protein contents, encoded by 15 genes located on different chromosome of rice genome (Kawakatsu et al. 2008). It is accumulated in irregularly-shaped protein bodies II (PB-II) derived from the protein storage vacuole along with GLO, as 57 kDa precursor (Kim et al. 2013). The GLUs are further divided into subfamilies: GluA, GluB, GluC, and GluD depending on resemblance in amino acid (Kawakatsu et al. 2008) with high nutrition value for human’s diet (Zhang et al. 2008). As, GLU is a major SSP of rice grain, any modification may definitely cause significant influence on grain quality. Several studies have been conducted to improve the nutritional quality of rice through altering SSPs (Yamuangmorn et al. 2021; Majumder et al. 2019).

Mutation breeding and transgenic approaches have been employed successfully to significantly reduce the GLU content with no compromise on total protein contents (Nagamine et al. 2011). However, these techniques holds several limitations e.g., time consuming, regulations measures and biosafety concerns. The advent of modern tools especially CRISPR/Cas9 system has opened plethora options for plant breeders to utilize minimum time for crop improvement programs (Fiaz et al., 2019). The CRISPR/Cas9 genome editing technology developed during 2013 has proved to be an effective and widely utilized technique in plants moreover, generating targeted mutation in wide array of cells and organisms (Barman et al. 2019). The system has been shown to be effective in cereals for the development of powdery mildew resistant *Triticum aestivum* (Wang et al. 2014), glutinous maize and *Oryza sativa* (Chilcoat et al. 2017; Zhang et al. 2018), *TGMS* in both *Oryza sativa* and *Zea mays* (Barman et al. 2019; Li et al. 2017), fragrant (Shao et al. 2017), herbicide resistant (Sun et al. 2016), nitrogen use efficiency (Li et al. 2018), bacterial blast resistant (Oliva et al. 2019), Low-cadmium (Yang et al. 2019), increased *β*-Carotene (Endo et al. 2019), drought resistance (Lou et al. 2017) germplasm have developed through editing *Waxy*, *OsTMS5*, *ZmTMS5*, *Badh2*, *ALS*, *NRT1.1B*, *SWEET*, *OsNRAMP5*, *Osor* and *SnRK2* genes, respectively.

From the past few decades the functional characterization of various genes i.e., *qPC1*, encoding a putative amino acid transporter OsAAP6, *gpa3*, *Osvpe1*, and *OsRab5a* have shown favorable results however, their cloning is difficult. In current era researchers along with food nutritionist working together to optimizing the nutritional level of cereals through integration of biotechnological approaches with traditional breeding. In present study, we conducted targeted mutagenesis of *Glu-B* (*LOC-Os02g15070*) gene in non-basmati rice PK386 cultivar background to generate mutant with reduced GLU content. The developed mutant can further be utilized in breeding programs to enhance the nutritional quality of rice. The reduced GLU content rice can proved to be a healthy diet for patients allergic to high GLU contents.

## Materials and methods

### Vector construction and rice transformation

The target sites (5′-GTTCGAAGAACATCTTTGATGG-3′ and 5′ CATTAGCAGTGGAGTAGCAAGG-3′) consist of a protospacer adjacent motif (PAM) lying within the *Glu-B* coding sequence *(LOC-Os02g15070)*. A BlastN search was conducted to ensure the uniqueness of the site. The gRNA target sequence was inserted into the pCAMBIA1300 × 35 S::Cas9-*Glu-A* expression vector was constructed. The resulting CRISPR/Cas9 construct was introduced into cv. PK386 by agro-infection as per procedure mentioned by Hiei et al. (1994). After about two months of culture, transgenic regenerates were transferred to a growth cabinet. The sequences of the primers used in vector construction and identification are listed in Table 1.

### Plant materials and growing conditions

**Fig. 1:**
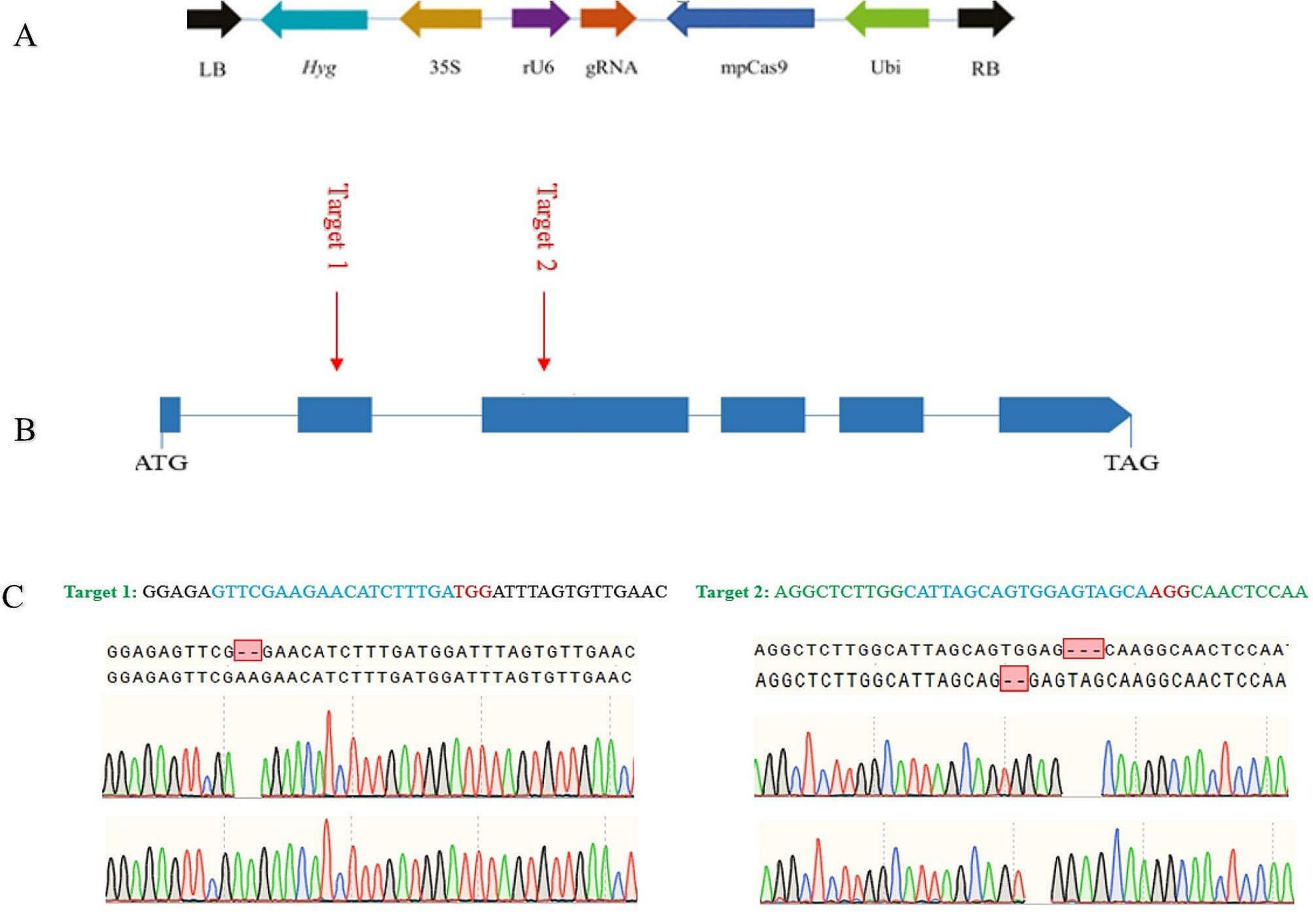
CRISPR/Cas9 mediated editing of *Glu-B* gene. **(A)**. The structure of the T-DNA region of the Cas9/guide RNA (gRNA) vector. Marker gene Hygromycin (Hyg) was driven by the CaMV35S (35 S) promoter whereas the gRNA was driven by the rice U6 promoter and the mpCas9 was driven by the Ubiquitin (Ubi) promoter. LB, Left border; RB, Right border. **(B)** The structure of *Glu-B* gene, two target sites were selected and sequenced in the mutant genotypes. **(C)** The *Glu-A* target site aligned with the *Glu-B-1* (a 2 nucleotides deletion) and the *Glu-B-2* (3 nucleotide deletion) mutant sequences in the same plant

To unrevealed on the function of *Glu-B*, a CRISPR/Cas9 vector containing a gRNA driven by the rice U6 promoter (Fig. 1-A) and carrying two target sites form the 1st and 2nd exon of *Glu-B* was constructed (Fig. 1-B). The plasmid was then inserted into wild-type PK386 calli via Agrobacterium- mediated transformation. Plants of T_0_ and T_1_ generation were grown in greenhouse and normal field condition maintaining normal agronomic practices to multiply seeds. T_2_ generation plants including wild-type cv. PK386 were cultivated in the field during the normal rice growing season under greenhouse condition at experimental facility, (24.7222° N, 46.6259° E) King Saud University, Riyadh Saudi Arabia. The seeds were harvested on maturity and stored at 37 °C for three month to evaluate the grain quality parameters.

### Mutant detection and analysis

Genomic DNA was extracted from the leaves of transformed plants using the sodium dodecyl sulfate (SDS) method (Dellaporta et al. 1983). Polymerase chain reaction (PCRs) amplifications were performed using primer pairs which generated an amplicon harboring the target site, and the resulting amplicons were sequenced using the Sanger method. Mutations were identified by comparing the amplicon sequences derived from putative transgenic and cv. PK386 templates. Homozygosity/heterozygosity for a mutated sequence was inferred from the chromatogram trace. T_1_ segregants homozygous for a *glu-B* mutation were screened for the presence/absence of T-DNA using a PCR assay directed to the hpt sequence using the pC1300-Cas9 plasmids and cv. PK386 gDNA as positive and negative controls, respectively. The relevant PCR primers for these steps are listed in Table 1.

### Grain quality traits measurement

From each individual line fully filled grains were utilized to evaluate grain quality. Hulls were removed from 50 g of grains by using Satake testing husker (THU-35 A Satake Engineering, Japan) and debranned with a McGill number 2 mill (seedburo Equipment, U.S.A.). Milled rice flour samples were obtained by grinding milled rice grains to pass through a 0.42 mm screen on an Udy cyclone mill (Cyclotec 1093 sample mill, Tecator, Sweden). The milled flour samples were sieved through a 100-mesh sieve to get uniform granule size. The GLU was measured using the micro-Kjeldahl pre-treatment method with some minor modifications (Chinese Bureau of Standardization, 1982). The GLU contents were prepared from rice flour based on the method of (Kumamaru et al. 1988) with minor modifications; The milled rice flour of 1.5 g with three repeats of each line was weighted for all fractions of protein separately, 0.1 M NaOH was used as extraction buffer for GLU. Moreover, the other grain quality i.e., GLO (%), albumin (%), PRO (%), total starch (%), amylose content (%), gel consistenct (mm), gelatinization temperature (oC), total sugar content (%) were measured utilizing standard protocols and recommended extraction buffers e.g., 0.5 M NaCl was used as extraction buffer for GLO; ddH20 was used as extraction buffer for ALB, and 70% n-propanol was used as extraction buffer for PRO (Kumamaru et al. 1988). Whereas, the agronomic traits i.e., plant height (cm), number of tillers, panicle length (cm), seed setting (%), seed length (cm), seed with (cm), seed thickness (cm) and 1000-grain weight (g) were recorded as per standard procedure.

### Scanning electron microscopy

The brown rice of wild-type and the *glu-B* mutant was cut transversely with the back of a knife, and the ruptured transverse surface was coated with gold to prepare samples. The ruptured transverse surface was observed by scanning electron microscope (SEM) which was performed as described previously using a HITACHI S-3400 N scanning electron microscope (http://www.hitachi-hitec.com) (Kang et al. 2005). For analyzing the development of compound starch granules, transverse sections (approximately 1 mm in thickness) of wild-type and *glu-B* endosperms at 30 DAF were used to make samples of semi-thin sections. Samples were treated as described by (Peng et al. 2014). Semi-thin Sect. (800 nm) were stained with I_2_-KI for 5 s and subsequently examined under a light microscope (Nikon Eclipse 80i; http://www.nikon.com).

### Gene expression analysis

Real-time qRT-PCR analysis was conducted using SYBR Premix EX TagTM (TaKaRa, Dalian, China) in a volume of 20 µl in a Bio-Rad CFX96 real-time PCR detection system3. The PCR parameters were as follows: 94 °C for 3 min, followed by 40 cycles of 94 °C for 15s, 60 °C for 20 s, and 72 °C for 20s. The wheat β-actin gene was used as a reference gene (Hu et al. 2013). Each amplification reaction was repeated three times. Validation experiments were performed to demonstrate that the amplification efficiency of the TaARGOS-specific primers was approximately equal to the amplification efficiency of the endogenous reference primers. Quantification of the target gene expression was carried by the comparative CT method (Livak and Schmittgen 2001). The primers employed for qRT-PCR are listed in Supplementary Table 1.

### GUS histo-chemical staining

The ∼ 2-kb putative promoter region of *Glu-B* (upstream of ATG) was amplified by PCR and cloned into the EcoRI/NcoI sites of pCAMBIA1305. The resultant construct was transformed into PK386 calli and independent lines of positive T_1_ transgenic progeny were used to detect GUS activity. Tissues and half-cut seeds were submerged in GUS staining solution (10mM EDTA, 0.1% Triton X-100, 1mM 5-bromo-4-chloro-3-indoyl- b-D-glucuronide, 100mM sodium phosphate (pH 7.0), 2.5mM K4Fe(CN)6 and 2.5mM K3Fe(CN)6 at 37 °C for 12–15 h. After incubation, tissues were discolored several times in pure ethyl alcohol.

### Statistical analysis

All data were analyzed using Excel 2016.

## Results

### Mutation of *Glu-A* via CRISPR/Cas9

A total of 60 T_0_ transgenic plants were ultimately obtained. Sequencing analysis of the *Glu-B* genomic locus in each T_0_ transgenic plant was performed to determine whether the targeted mutation had occurred. Results showed that 25 independent plants displayed an edited sequence. Five types of homozygous and two heterozygous mutations at both target site were found: 2 nucleotides deletion in target site 1 along with 3 and 2 nucleotide deletion in target site 2 was found in T_0_ mutant plants (Fig. 1-C).

### The rice *glu-B* mutant displays reduction in GLU content and physio-chemical properties

**Fig. 2:**
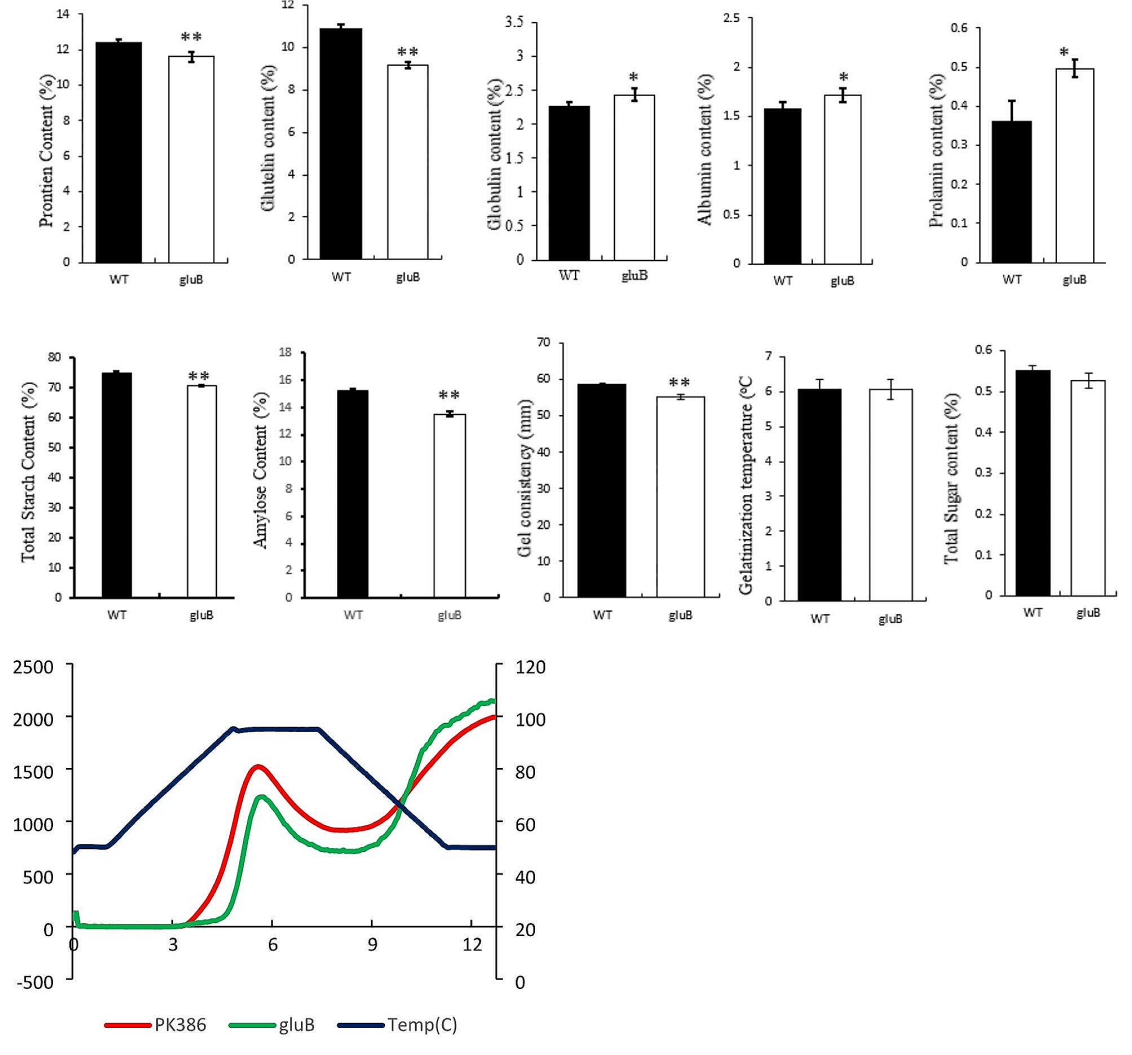
The performance of cv. PK386 (WT) and *glu-A* mutant. **(A)** Protein content (%), **(B)** Glutelin content (%), **(C)** Globulin content (%), **(D)** Albumin content (%), **(E)** Prolamin content (%), **(F)** Total starch content (%), **(G)** Amylose content (%), **(H)** Gel consistency (mm), **(I)** Gelatinization temperature (^o^C), **(J)** Total Sugar content (%), **(K)** Paste viscosity; red curve shows PK386, Green curve shows *glu-A* mutant. Data are given as means ± SD from three replicates. Statistical comparisons were performed using Student’s t-test; all data were compared with WT (*P < 0.05, **P < 0.01)

The mutant shown significant variation for SSPs in comparison to wild type (WT) plant. The results displayed the significant reduction in total protein content (PC) along with highly significant reduction in GLU, however GLO, ALB and PRO contents displayed highly significant to significant increase, respectively. The physico-chemical characteristics, total starch content, amylose content, gel consistency and paste viscosity displayed highly significant reduction compared to WT whereas, gelatinization temperature and total sugar content showed no differences compare with WT (Fig. 2). It can also assumed the targeted gene holds influence for both SSPs and starch related properties.

### Agronomic related traits and grain appearance quality in *glu-A* and WT

**Fig. 3:**
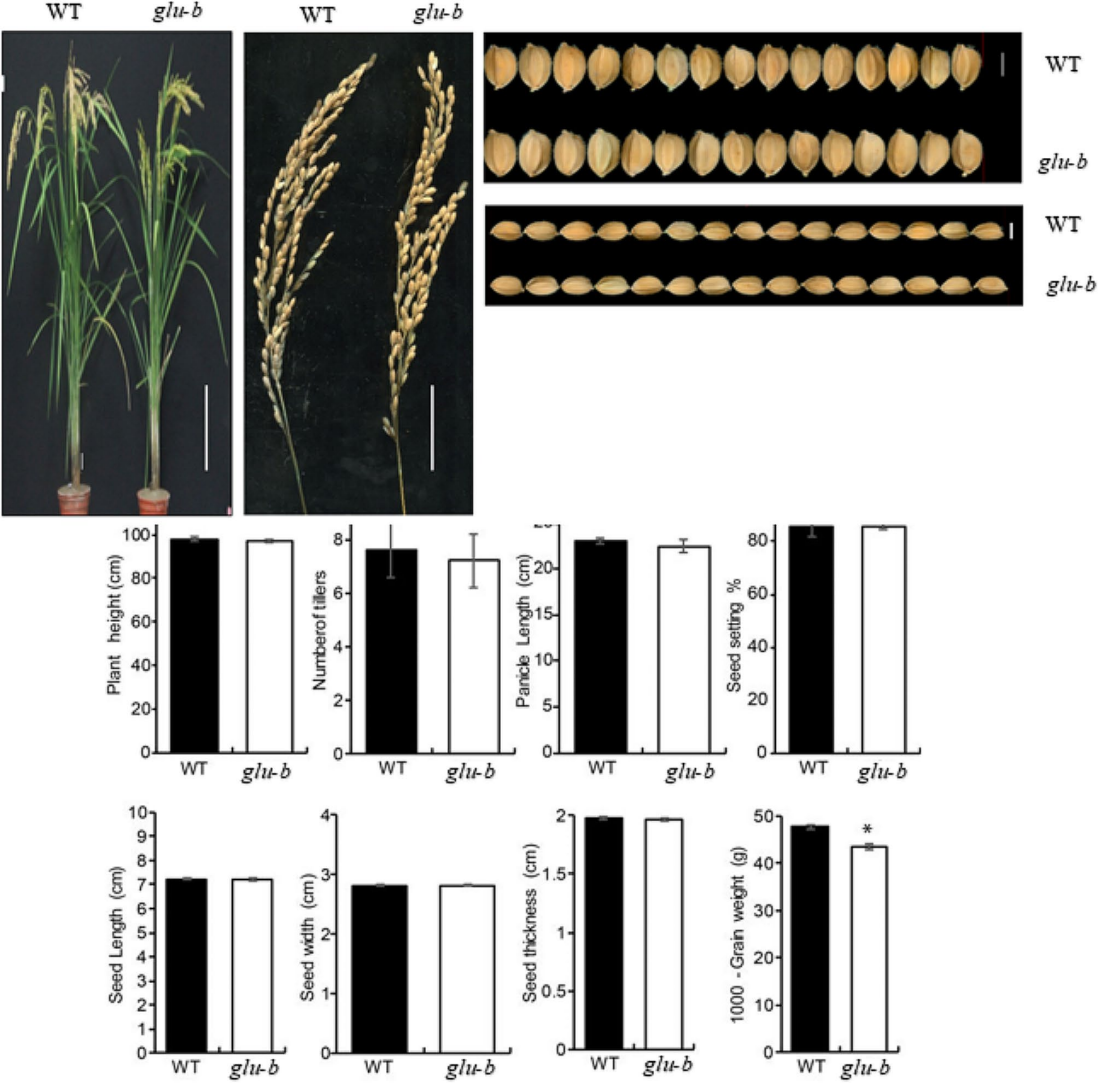
The agronomic and appearance quality related traits for WT and *glu-A*. Data are given as means ± SD from three replicates. Statistical comparisons were performed using Student’s t-test; all data were compared with WT (*P < 0.05, **P < 0.01)

The yield and yield related traits of WT plants and *gluA* mutant were compared. Results displayed non-significant variation for all agronomic related traits in comparison to WT except for 1000-grain weight. The 1000-grain weight might be effected owing to the chalkiness in *gluA* mutant (Fig. 3). Furthermore, the presence of chalkiness influenced the cooking and eating quality of rice.

### Scanning electron microscopy for WT and *glu-A* mutant

**Fig. 4:**
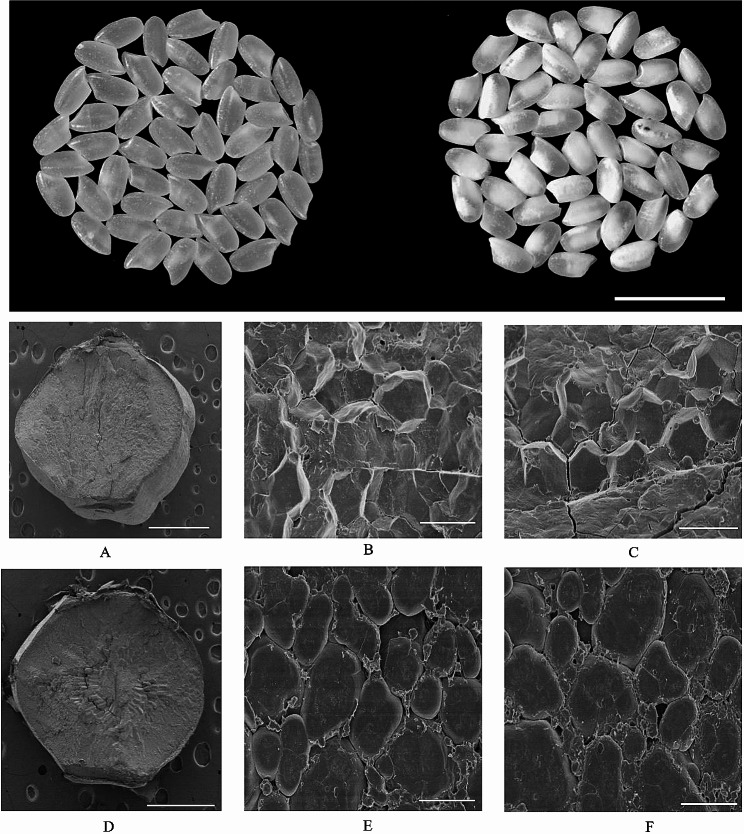
Scanning electron microscopy images for WT and *glu-b* (T_2_ generation). **(A)** Mature endosperm of WT. **(B)** Central region of mature endosperm in WT **(C)** Peripheral region in mature endosperm in WT. **(D)** Mature endosperm of *glu-b* mutant. **(E)** Central region of mature endosperm in *glu-b* mutant. **(F)** Peripheral region in mature endosperm in *glu-b* mutant

The scanning electron microscopy (SEM) of the cross-sections of mature endosperm displayed variation among starch granules in the central region of both wild type and mutant. The wild type showed closely packed, regular polyhedron shaped structure (Fig. 4-A, B and C) whereas, mutant endosperm displayed loosely packed, irregular shaped granule structure (Fig. 4-D, E and F).

### Expression analysis of WT and *glu-b* mutant

**Fig. 5:**
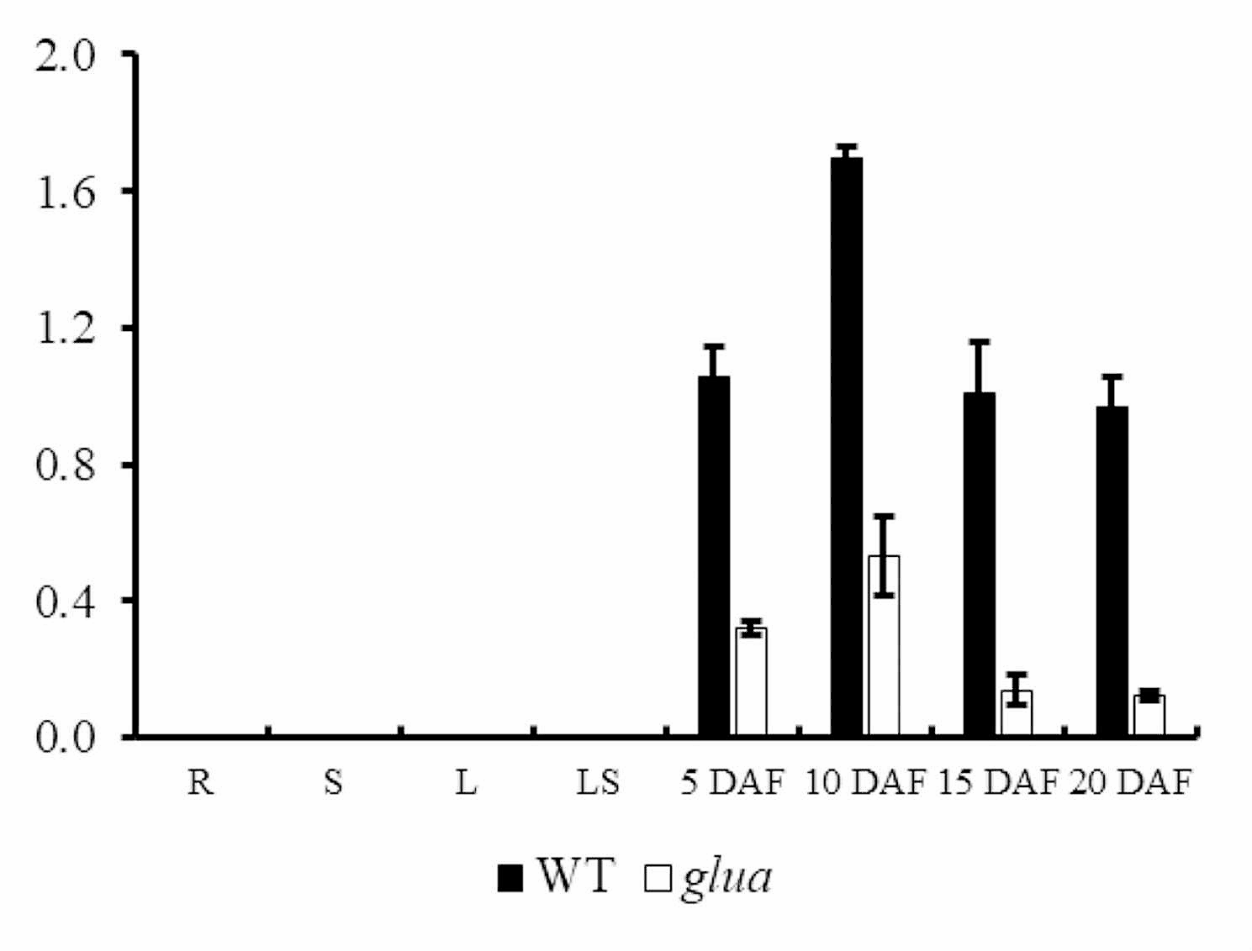
*glu-b* expression in cv. PK386 and T_2_ generation of *glu-b* mutant. Transcript abundance as estimated by qRT-PCR in the R (roots), S (stem), L (leaves), LS (leaf sheath) at 5, 10, 15 and 20 DAF (days after flowering). Data are given as means ± SD from three replicates

The expression analysis of *glu-b* in cv. PK386 and mutants plants grown at field condition were analyzed for the abundance of mutant *glu-b* transcript in R (roots), S (stem), L (leaves), LS (leaf sheath) at 5, 10, 15 and 20 DAF (days after flowering). It was found *Glu-A* transcript was considerably higher in WT comparison to the mutants (Fig. 5a). At the protein level, SDS page indicated that the abundance of *glu-b* in the seed of PK386, whereas protein signals were barely detected in mutants (Fig. 5b, c). Thus the mutant can be considered as viable candidate to be used for low gluten rice breeding program.

### Sub-cellular localization of *glu-b* mutant

**Fig. 6:**
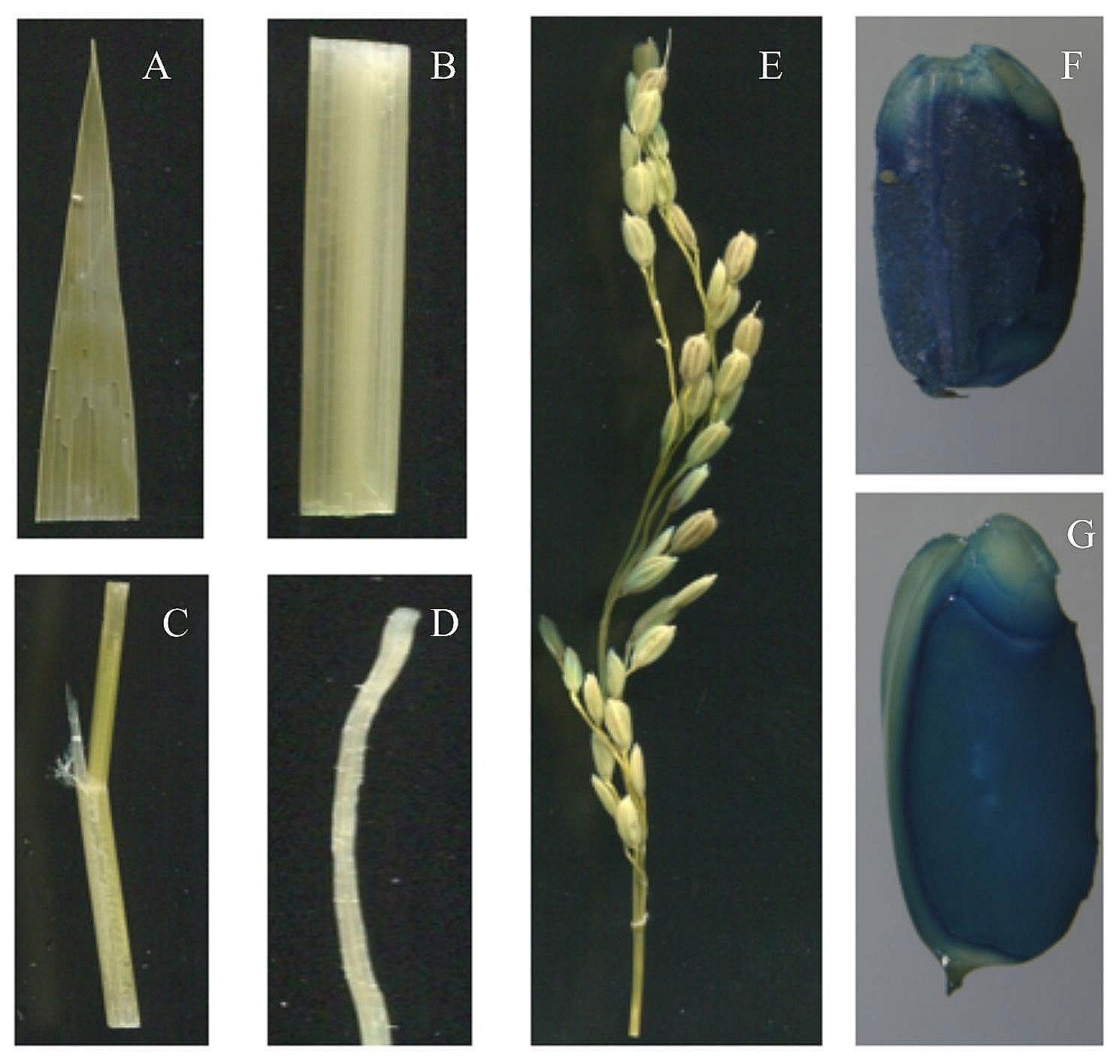
GUS expression in **A**, leaf **B**, leaf sheath **C**, stem **D**, root **E**, panicle **F**, brown rice and **G**, cut brown rice. Bars: 2 mm

The expression pattern of *Glu-B* gene was further investigated *Glu-B*: GUS transgenic rice plants using a *β-glucuronidase* (GUS) reporter gene under the control of the *Glu-B* gene promoter. The results were consistent as of qRT-PCR expression analysis results, no GUS activity was recorded for leaf, leaf sheath, stem and root (Fig. 6. A, B, C, D), low GUS activity was recorded for panicle (Fig. 6, E) whereas, strong GUS activity was recorded for mature endosperm (brown rice and cut brown rice) (Fig. 6. F & G). These results indicated that *Glu-B* gene is mainly expressed in the mature endosperm of rice plant.

## Discussion

Rice is largest source of providing calories and nutrition to approximately more than half of the world population (Barman et al. 2019). The rice grain quality is multifaceted trait and improvement in single trait does not confer the overall improvement in grain quality. The nutritional quality improvement could be only possible by improving SSPs contents however, the genetic framework of SSPs is complex, polygenic and consist of mixture of polypeptide which are further polymorphic in nature. The induced mutations in a single or few genes may have no significant influence on SSPs composition (Shewry 2007; Kawakatsu et al. 2010). Moreover, the nature of being major or minor gene/s, epistatic effects and environmental interaction is also not contain considerable impact. Based on, nature of SSPs researchers are came up with conclusion to employ both conventional and modern molecular based strategies to harness concentration of SSPs in rice grain (Yang et al. 2008). Scientist around the globe are working to develop low GLU rice grain owing to its multiple health benefits. During 1993, Japanese researcher developed low GLU protein mutant NM67 via employing ethyleneimine mutagenesis which further utilized to developed first low GLU Japonica rice variety LGC-1 and subsequently, two more varieties were developed LGC-Katsu and LGC-Jun from a cross between LGC-1 and a mutant line of Koshihikari (Nishimura et al. 2005). The variety LGC-1 was later introduced in China and widely employed in various breeding program on developing low GLU rice (Cai et al. 2015). The SSPs are classified into four fractions ALB, GLO, PRO, and GLU among these glutelin makes approximately 80% of SSPs and encoded by 15 genes from four sub-families GluA, GluB, GluC, and GluD (Chen et al. 2022). Based on the genetic architecture and complicated nature of SSPs require comprehensive research handling the limitation of germplasm and genes redundancy for developing low GLU rice.

Several published literature has documented the efforts to down-regulate SSPs in rice utilizing RNA-silencing (Kawakatsu et al. 2010), RNA-interference (Kim et al. 2012; Cho et al. 2016) and CRISPR/Cas9 (Chen et al. 2022) has been successfully employed to target gene/s controlling total protein a fractions of proteins in rice. In present investigation, both target sites in exon 2 and 3 displayed deletion of base pairs which may change the amino acid sequence and later protein structure as well. The deletion of base pairs showed phenotypic changes in *glu-b* mutant plants once recorded the physio-chemical properties compared to wild type. Previous literature has displayed the reduced expression in gene/s controlling any fraction of protein correlated with an increase in expression of other SSPs (Qian et al. 2018). Our results are in complete agreement to these findings where we found, the *glu-b* mutant displayed reduction in total protein and GLU contents whereas, ALB, GLO and PRO shown elevation in their concentration. However, further investigations are required to fully explore the pathway controlling the compensatory alternations in the concentration of SSPs other than GLU. The plants both wild type and *glu-b* mutant also displayed normal growth and development and there were no significant difference observed for overall grain length and width. Furthermore, the *glu-b* mutant displayed no significant difference for agronomic traits being recorded expect for 1000-grain weight.

The morphology of brown rice grain was also similar both in wild type and *glu-b* mutant expect highly significant degree of chalkiness in mutant plant grains. The SSPs and starch are the main ingredients of rice grain (Yang et al. 2019). Their biosynthesis and deposition in rice endosperm is regulated in coordination through complex transcriptional and translational mechanism (Biselli et al. 2015). Moreover, the defects during biosynthesis and accumulation of SPPs may cause positive or negative consequences on starch or starch related properties of rice grain. Therefore, in *glu-b* mutant grains we observed higher degree of chalkiness. The grain chalkiness is considered the degree of opaque endosperm, usually composed of irregular starch granules or presence of abnormal protein bodies (Li et al. 2014). To explore the mechanism of chalkiness, the cross sectional study of mature endosperm of both wild type and *glu-b* mutant displayed divergent results, mutant endosperm showed irregular, round sphere and loosely packed starch granules with highly significant reduction in total starch and amylose contents which are considered negative traits influencing eating and cooking quality of cooked rice grain. To understand the expression of *Glu-B* gene both in wild type and mutant the expression analysis was undertaken and it was observed the *glu-b* mutant has displayed lower transcript level at 5, 10, 15 and 20 DAF which could be the possible reason for lower concentration of GLU in mutant lines. Furthermore, the sub-cellular localization results confirmed the expression analysis findings, the presence of GLU-B was observed in mature endosperm through GUS activity. Based on these findings, it can be observed the CRISPR/Cas 9 system could be useful tool being employed to effectively develop germplasm having significant breeding importance.

## Conclusion

In summary, we demonstrated the application of CRISPR/Cas9 genome editing system for developing low GLU mutant with promising agronomic attributes, including yield. Moreover, the study could be utilized as foundation knowledge for studying gene families controlling SSPs in cereals and regulatory mechanisms controlling complex network of both SSPs and starch properties in rice grains. We envision that present study could open plethora option for breeding commercial varieties having variant glutelin contents, suitable for patients unable to consume high glutelin rice.

## Electronic supplementary material

Below is the link to the electronic supplementary material.

**Supplementary Material 1**: **Supplementary table 1** Sequence of primers used in present study.

## Data Availability

Data are contained within the article and Supplementary Materials.
