# Supplementary material for: Application of CRISPR/Cas9 system to knock out GluB gene for developing low glutelin rice mutant
Source: Bot Stud. 2024 Sep 3;65:27. doi: 10.1186/s40529-024-00432-0 (PMC11371991; doi:10.1186/s40529-024-00432-0)
Supplement: Supplementary file 1 — Supplementary Material 1: Supplementary table 1 Sequence of primers used in present study. [file 40529_2024_432_MOESM1_ESM.docx]

| **Primer Name** | **Primer sequence** | **bp length** |
| --- | --- | --- |
| **Target amplification primer** | | |
| 2-1-F | ggcaGTTCGAAGAACATCTTTGA | 23 |
| 2-1-R | aaacTCAAAGATGTTCTTCGAAC | 23 |
| 2-2-F | ggcaCATTAGCAGTGGAGTAGCA | 23 |
| 2-2-R | aaacTGCTACTCCACTGCTAATG | 23 |
| **Expression analysis primer** | | |
| qRT-F | GTGCTAACCAGCTTGATCCT | 20 |
| qRT-R | CAAGAACAATTTCTCCGCGT | 20 |
| **Sub-cellular localization primer** | | |
| 55630-GUS-F | ***CCATGATTACGAATTC***GACCAGTCCACGACCTCAA | 35 |
| 55630-GUS-R | ***CTCAGATCTACCATGG***TAATAAAAGGATATAAAAG | 35 |

**Table S1. List of Primers**
